# Chromatin remodeling protein HELLS is upregulated by inactivation of the RB-E2F pathway and is nonessential for osteosarcoma tumorigenesis

**DOI:** 10.18632/oncotarget.25953

**Published:** 2018-08-24

**Authors:** Stephanie C. Wu, Claudia A. Benavente

**Affiliations:** ^1^ Department of Developmental and Cell Biology, University of California, Irvine, CA 92697, USA; ^2^ Department of Pharmaceutical Sciences, University of California, Irvine, CA 92697, USA

**Keywords:** osteosarcoma, HELLS, RB, E2F, cancer

## Abstract

Osteosarcoma is the most common primary bone malignancy in children and adolescents. Among the various molecular mechanisms implicated in osteosarcomagenesis, the RB-E2F pathway is of particular importance as virtually all cases of osteosarcoma display alterations in the RB-E2F pathway. In this study, we examined the transcription factor E2F family members that are associated with increased malignancy in *Rb1*-null osteosarcoma tumors. Using genetically engineered mouse models of osteosarcoma, we found that loss of activator E2Fs, E2F1 and E2F3, significantly delays tumor progression and increases the overall survival of the *p53/Rb1*-deficient osteosarcoma mouse model. We also studied the role of helicase, lymphoid specific (HELLS), a chromatin remodeling protein identified as a critical downstream effector of the RB-E2F signaling pathway in various cancers. In this study, we confirmed that the RB-E2F pathway directly regulates *HELLS* gene expression. We also found that *HELLS* mRNA is upregulated and its protein overexpressed in osteosarcoma. Using loss-of-function assays to study the role of HELLS in human osteosarcoma, we observed that HELLS has no effect on tumor proliferation and migration. Further, we pioneered the study of *Hells* in developmental tumor models by generating Hells conditional knockout osteosarcoma mouse models to examine the role of HELLS in osteosarcoma tumor development. We found that loss of *Hells* in osteosarcoma has no effect in tumor initiation and overall survival of mice. This suggests that while HELLS may serve as a biomarker for tumorigenesis and for RB-E2F pathway status, it is unlikely to serve as a relevant target for therapeutics in osteosarcoma.

## INTRODUCTION

Osteosarcoma is a mesenchymal tumor that is histologically characterized by the presence of malignant mesenchymal cells and the production of a bone stroma. Osteosarcomas are commonly characterized by an appendicular primary tumor with a high rate of metastasis to the lungs [1]. The most common genetic findings in osteosarcoma are the dysregulation of the *TP53* and *RB1* tumor suppressor genes [2, 3]. Loss-of-function mutations of the *RB1* gene in osteosarcoma are associated with poor therapeutic outcome, as defined by increased mortality, metastasis, and poor response to chemotherapy [4–8]. Unfortunately, clinical outcomes for osteosarcoma patients have not substantially improved in over 30 years. As a result, the 5-year overall survival rate has remained stable at ~65% in case of local disease and ~20% for patients with metastatic disease [9–12].

The *RB1* gene encodes the tumor suppressor protein RB. RB forms a transcriptional repression complex with the E2F family of transcription factors and thereby negatively regulates G_1_/S transition during the cell cycle through the suppression of E2F target genes. There are six E2F family members that bind to the RB family and are classified as activator E2Fs (aE2Fs: E2F1, E2F2 and E2F3a) and repressor E2Fs (rE2Fs: E2F3b, E2F4, and E2F5) [13]. Of these, aE2Fs show preferential binding to RB protein. In response to growth stimuli, the cyclin D–CDK4/6 complex phosphorylates RB, relieving aE2Fs to facilitate the transcription of genes required for G_1_/S transition. In addition to E2Fs, RB associates with a large number of nuclear proteins, including a variety of chromatin-associated proteins that have diverse activities [14–16]. *RB1* mutations are present in up to 70% of osteosarcoma cases [17]. Furthermore, multiple studies have shown that *RB1* loss is correlated with poor prognosis for patients with osteosarcoma [4–8]. Specifically, loss of *RB1* function is associated with increased risk of osteosarcoma metastasis and poor response to chemotherapy, compared with osteosarcoma patients with intact *RB1* [5, 18, 19]. A previous study performed in retinoblastoma, a childhood cancer caused by bi-allelic loss of *RB1*, indicated that retinal tumorigenesis was driven by E2F1- and E2F3-transcribed genes following the lack of transcriptional repression due to RB loss [20].

HELLS (helicase, lymphoid specific; also known as LSH) is a protein that belongs to the SNF2 family of chromatin-remodeling ATPases [21]. HELLS is critical for normal development of mammals by establishing DNA methylation patterns across the genome [22]. In retinoblastoma, HELLS was identified as a critical contributor of Rb-mediated tumorigenesis [20]. In addition to retinoblastoma, several reports have shown that HELLS overexpression contributes to malignant progression including renal cell carcinoma, gliomas, prostate cancer, melanoma, and nasopharyngeal carcinoma [23–27]. In astrocytomas and glioblastomas, upregulation of E2F1 correlated with increased HELLS expression and increased along with tumor grades [24]. However, the role of HELLS in tumor initiation, particularly in osteosarcoma, has never been examined.

In this study, we generated a series of genetically engineered mouse models of osteosarcoma to study the role of E2Fs in tumor development. We focused on E2F1 and E2F3, two aE2F previously described as critical for Rb-mediated tumorigenesis, and E2F5 as a rE2F control [20]. Similar to what was observed in retinoblastoma, we found that E2F1 and E2F3 contribute to increased malignancy associated with loss of *Rb1*. Since HELLS was identified as aE2F target gene and critical contributor of tumorigenesis in retinoblastoma, we evaluated the role of HELLS in osteosarcoma tumorigenesis. We demonstrated that activator E2Fs, E2F1 and E2F3, directly regulate HELLS expression. We also showed that RB-E2F pathway inactivation, present in most osteosarcomas, results in increased HELLS expression. Further, we evaluated the role of HELLS in osteosarcoma cell survival, proliferation, migration, and tumorigenesis both *in vitro* and *in vivo*. We showed that, unlike what has been observed in other malignancies, targeting HELLS in human osteosarcoma has a modest effect in osteosarcoma survival and no effect on migration. Further, using a *Hells* conditional knockout mouse model, we found that loss of *Hells* has no effect on osteosarcoma tumor incidence and overall survival. This suggests that HELLS is not critical for tumor initiation and progression in osteosarcoma.

## RESULTS

### RB regulates tumor progression through E2F1 and E2F3

**Figure 1:**
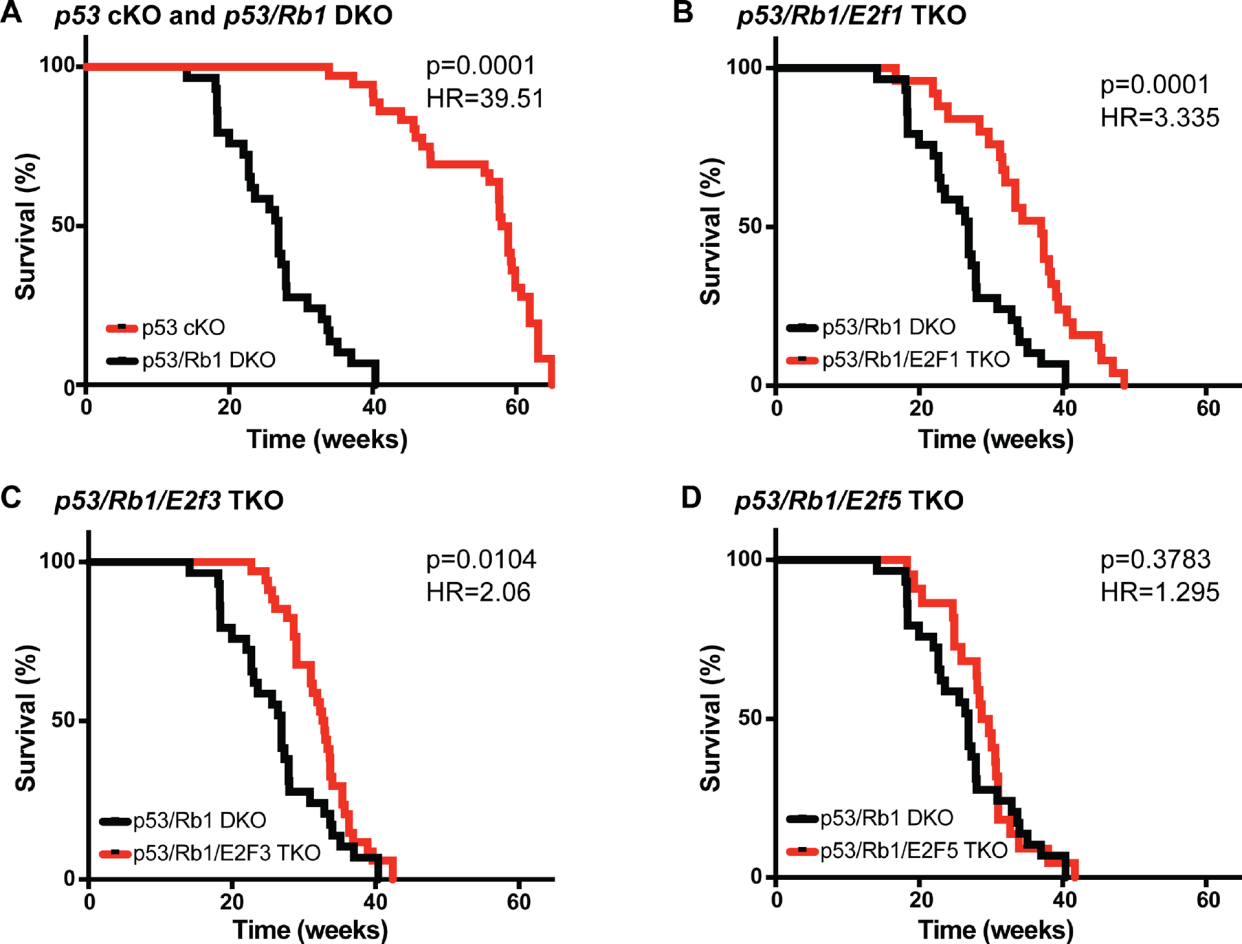
Loss of E2f1 or E2f3 increases survival in osteosarcomas bearing Rb1 mutations (**A**–**D**) Kaplan–Meier curves showing the survival of osteosarcoma-prone mouse models. (A) Mice bearing *Rb1* mutations p53/Rb1 DKO: *Osx-cre*; *p53^lox/lox^*; *Rb1^lox/lox^* (black, *n* = 29) have significantly shorter lifespan compared to p53 cKO: *Osx-cre*; *Tp53^lox/lox^* (red, *n* = 35). This survival time was significantly increased in (B) *p53/Rb1/E2f1* TKO: *Osx-cre*; *p53^lox/lox^*; *Rb1^lox/lox^*; E2f1−/−(red, *n* = 25) and (C) *p53/Rb1/E2f3* TKO: *Osx-cre*; *p53^lox/lox^*;*Rb1^lox/lox^*; *E2f3^lox/lox^* (red, *n* = 34); but not in (D) *p53/Rb1/E2f5* TKO: *Osx-cre*; *p53^lox/lox^*;*Rb1^lox/lox^*;*E2f5^lox/lox^* (red, *n* = 22). Mantel-Cox test and Mantel-Haenszel hazard ratio (HR) were used for curve comparisons.

Recombination of *Tp53* in conditional knockout mice driven by osterix-cre recombinase (*Osx-cre*), a transgene that is actively expressed in more differentiated pre-osteoblasts, results in osteosarcoma with high penetrance (*p53* cKO: *Osx-cre; p53^lox/lox^*) [28]. This disease model is potentiated by the loss of *Rb1* (*p53/Rb1* DKO: *Osx-cre; p53^lox/lox^; Rb1^lox/lox^*), recapitulating the negative impact of *RB1* mutations in human osteosarcoma [28]. Previous studies have shown that loss of *E2f1* and *E2f3*, but not *E2f5*, prevents retinoblastoma formation in *Rb1*-deficient mice [20, 29]. We hypothesized that in *Rb1*-deficient mice, aE2Fs (E2F1 and E2F3) constitutively facilitate transcriptional activation of downstream targets that associate with poor outcome. To determine if loss of these E2F family members can revert the increased malignancy associated with loss of *Rb1*, we developed *Osx-cre; p53^lox/lox^; Rb1^lox/lox^; E2f1*^−/−^ (*p53/Rb1/E2f1* TKO), *Osx-cre; p53^lox/lox^;Rb1^lox/lox^;E2f3^lox/lox^* (*p53/Rb1/E2f3* TKO), and *Osx-cre; p53^lox/lox^;Rb1^lox/lox^;E2f5^lox/lox^* (*p53/Rb1/E2f5* TKO) triple-knockout mice. To characterize the tumor incidence in these mouse models, we monitored mice weekly until advanced tumor burden required euthanasia. 100% (*n* = 35) of the *p53* cKO mice developed tumors by 65 weeks with a median survival of 58.45 weeks (Figure 1A). 100% (*n* = 29) of the *p53/Rb1* DKO mice developed tumors by 40.4 weeks with a median survival of 26.9 weeks (Figure 1). 100% (*n* = 25) of the *p53/Rb1/E2f1* TKO mice developed tumors by 48.6 weeks with a median survival of 37 weeks, significantly higher than *p53/Rb1* DKO mice (*p* = 0.0001; Figure 1B). 100% (*n* = 34) of the *p53/Rb1/E2f3* TKO mice developed tumors by 42.4 weeks with a median survival of 32.75 weeks, also significantly higher than *p53/Rb1* DKO mice (*p* = 0.0104; Figure 1C). In contrast, 100% (*n* = 22) of the *p53/Rb1/E2f5* TKO mice developed tumors by 41.7 weeks with a median survival of 29.2 weeks, which was not significantly different from *p53/Rb1* DKO mice (*p* = 0.3783) (Figure 1D). Further, we found no significant gender differences in any of these mouse models (Supplementary Figure 1).

### HELLS is overexpressed in both human and mouse osteosarcoma

**Figure 2:**
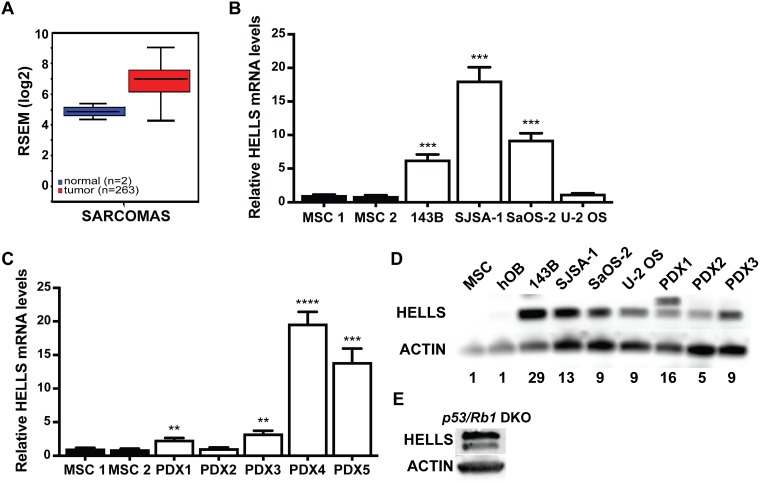
HELLS is overexpressed in human and mouse osteosarcoma (**A**) The Cancer Genome Atlas data of RNA-Seq by Expectation-Maximation (RSEM) values from 263 human sarcoma samples (red) compared to normal control (blue) show a 4.27-fold increase in *HELLS* mRNA expression. (**B**–**C**) RT-qPCR analysis of HELLS mRNA expression in (B) 143B, SJSA-1, SaOS-2 and U-2 OS osteosarcoma cell lines and (C) patient-derived xenografts (PDX1-5), normalized to mesenchymal stem cells (MSC1 and MSC2) (**D**) Western blot detection of HELLS in osteosarcoma cell lines and PDXs show increased protein levels compared to two lineage progenitor controls, MSCs and human osteoblasts (hOB). Actin used as loading control. Band intensities were quantified by densitometry and normalized to MSC. (**E**) Western blot detection of HELLS in a mouse *p53/Rb1* DKO tumor.

A previous study in retinoblastoma identified HELLS as a key target gene downstream of the RB-E2F signaling pathway that is overexpressed following the loss of RB, and contributes to tumorigenesis [20]. Given that *RB1* loss in osteosarcoma patients is associated with poor prognosis, we hypothesized that loss of RB potentiates osteosarcoma through transcriptional deregulation of chromatin remodeling genes including *HELLS*. Initial analysis of The Cancer Genome Atlas (TCGA) data set of 263 sarcoma tumor samples indicated that *HELLS* is upregulated by 4.27-fold compared to normal controls, making HELLS an attractive target to study in osteosarcoma (Figure 2A). Further analyses of HELLS gene expression using real-time qPCR in human osteosarcoma cell lines (143B, SJSA-1, SaOS-2, and U-2 OS) and patient derived xenografts (PDX1-5) showed a significant upregulation of *HELLS* mRNA level in most osteosarcoma samples when compared to human mesenchymal stem cells (MSCs) (Figure 2B–2C). At the protein level, HELLS was also highly overexpressed across all analyzed osteosarcoma subjects compared to MSCs and human osteoblasts (hOB) controls (Figure 2D). Increased HELLS protein expression was observed even in U-2 OS and PDX2, which showed no upregulation at the mRNA level. Further, HELLS protein overexpression was also observed in the *p53/Rb1* DKO osteosarcoma mouse model (Figure 2E). We also noted that some primary tumors, both in human and mouse, express a longer HELLS protein. Whether this is representative of post-translational modifications (HELLS can be phosphorylated, acetylated, ubiquitinated, methylated and sumoylated) or a HELLS isoform was not investigated.

### HELLS is a direct target of the RB-E2F signaling pathway in the osteogenic lineage

**Figure 3:**
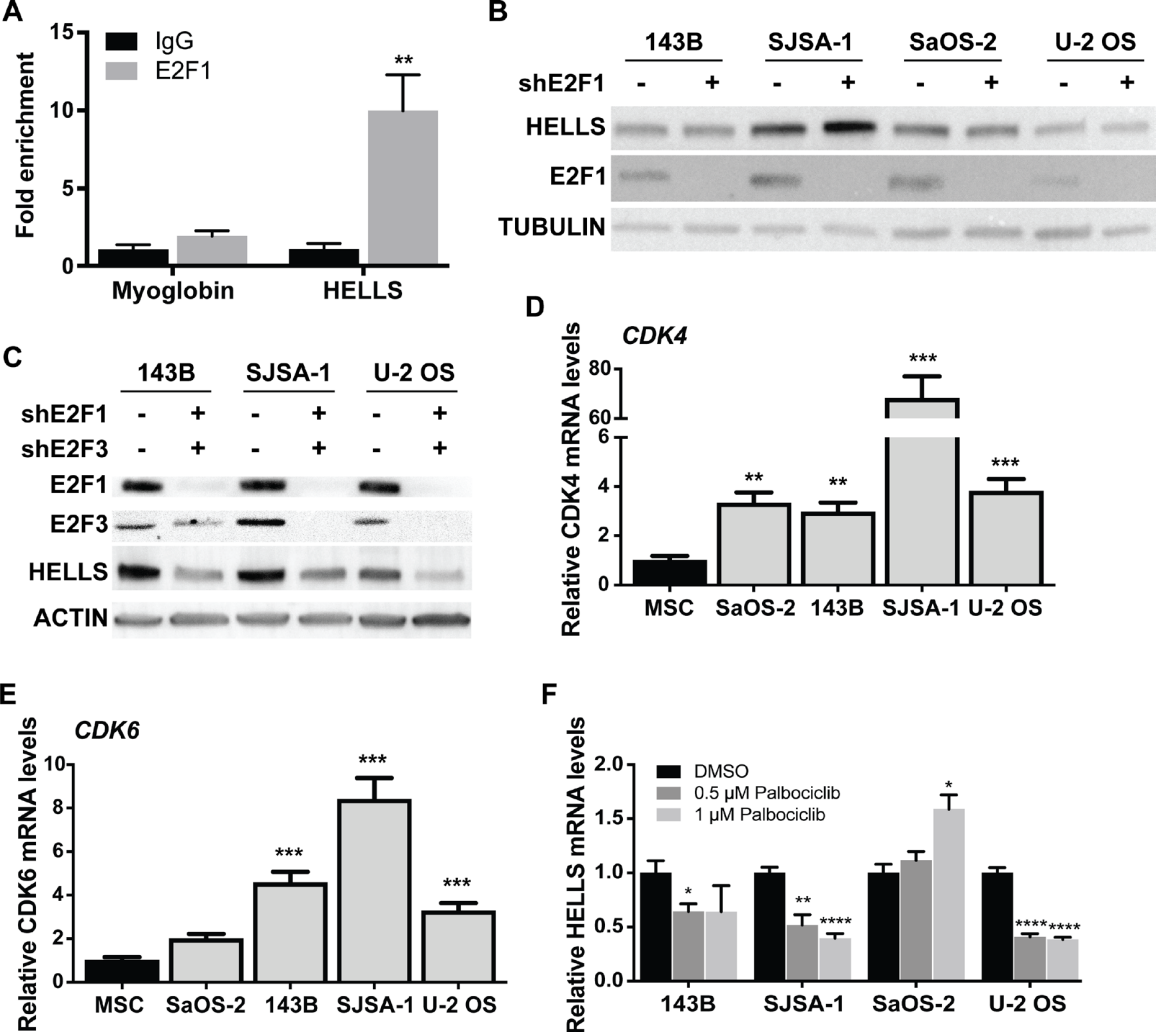
*HELLS* is a direct transcriptional target of the RB-E2F pathway (**A**) Chromatin immunoprecipitation (ChIP) assay reveals enrichment of E2F1 within *HELLS* promoter. Primers flanking the myoglobin promoter were used as negative control. Each point is mean ± s.d. of triplicate samples. (**B**–**C**) Western blot detection of HELLS protein level in osteosarcoma cells transduced with lentivirus containing shRNA against (B) E2F1 (shE2F1) show no significant change in HELLS expression, but (**C**) combination of E2F1 (shE2F1) and E2F3 (shE2F3) knockdown decreased HELLS protein levels. (**D**–**E**) RT-qPCR analysis of basal expression of (D) CDK4 and (E) CDK6 mRNA in different osteosarcoma cell lines normalized to mesenchymal stem cells (MSC). Each point is mean ± s.d. of triplicate samples. (**F**) RT-qPCR analysis of HELLS mRNA expression in osteosarcoma cell lines after Palbociclib-treated for 24 h, normalized to DMSO control cells. Each point is mean ± s.d. of triplicate samples. ^*^p < 0.0332, ^**^ *p* < 0.0021, ^***^p < 0.0002, ^****^p < 0.0001 by two-tailed *t* test.

Evidence of direct regulation of HELLS by the RB-E2F signaling pathway is limited to a single *in vitro* study in gliomas [24]. To confirm that the RB-E2F pathway transcriptionally represses HELLS, we performed chromatin immunoprecipitation analysis (ChIP) in the osteosarcoma lineage of origin using MSCs. Motif mapping using MotifMap revealed a putative E2F1 binding motif within the promoter region of HELLS, 97 bp upstream of the start codon [30]. PCR primers were designed to flank this area of putative E2F1 binding motif. As seen in Figure 3A, we observed enrichment of E2F1 at the consensus binding site at the HELLS promoter (Figure 3A).

To confirm direct transcriptional regulation of E2F1 on *HELLS* in human osteosarcoma, we acquired lentiviral vectors encoding shRNAs targeting E2F1 (shE2F1) and transduced the four human osteosarcoma cell lines in which we had previously observed HELLS overexpression (Figure 2D). Interestingly, we found that E2F1 knockdown was not effective at reducing HELLS protein levels in any of the osteosarcoma cell lines used (Figure 3B). To determine if other aE2F family members could compensate for the loss of E2F1, we acquired lentiviral vectors encoding shRNAs targeting E2F2 (shE2F2) and E2F3 (shE2F3). Knockdown of E2F2 or E2F3 using shE2F2 or shE2F3 alone did not affect HELLS protein expression (Supplementary Figure 2B); neither did the combination of shE2F1 and shE2F2 (Supplementary Figure 2A). However, combinatorial knockdown of E2F1 and E2F3 using shE2F1 and shE2F3 achieved a significant reduction in HELLS protein levels (Figure 3C).

Of the four osteosarcoma cell lines used in this study, SaOS-2 is the only one bearing an *RB1* mutation. To assess the mechanism through which the RB-E2F pathway is being deregulated in the osteosarcoma cell lines without *RB1* mutations, we analyzed *CDK4* and *CDK6* gene expression. *CDK4* and *CDK6* amplification and overexpression has been reported in some osteosarcomas, resulting in RB hyperphosphorylation [31]. Real time qPCR analysis of *CDK4* showed a significant increase in its transcription in all osteosarcoma cell lines (Figure 3D). A particular high expression of *CDK4* was observed in SJSA-1, a cell line with known *CDK4* amplification [32]. *CDK6* was also upregulated in the 3 osteosarcoma cell lines without *RB1* mutations: 143B, SJSA-1 and U-2 OS (Figure 3E). In order to confirm that increased CDK4 and CDK6 activity results in RB hyperphosphorylation and leads to overexpression of HELLS, we treated all four human osteosarcoma cell lines with CDK4/6 inhibitor, palbociclib [33]. Western blot analysis confirmed reduction in RB phosphorylation following palbociclib treatment (Supplementary Figure 2C). Real-time qPCR analysis of *HELLS* mRNA levels revealed significant decreases in *HELLS* expression upon palbociclib treatment, with the exception of the *RB1*-null cell line, SaOS-2, which served as a negative control (Figure 3F).

### HELLS has limited effect on human osteosarcoma cell proliferation and no effect on migration

**Figure 4:**
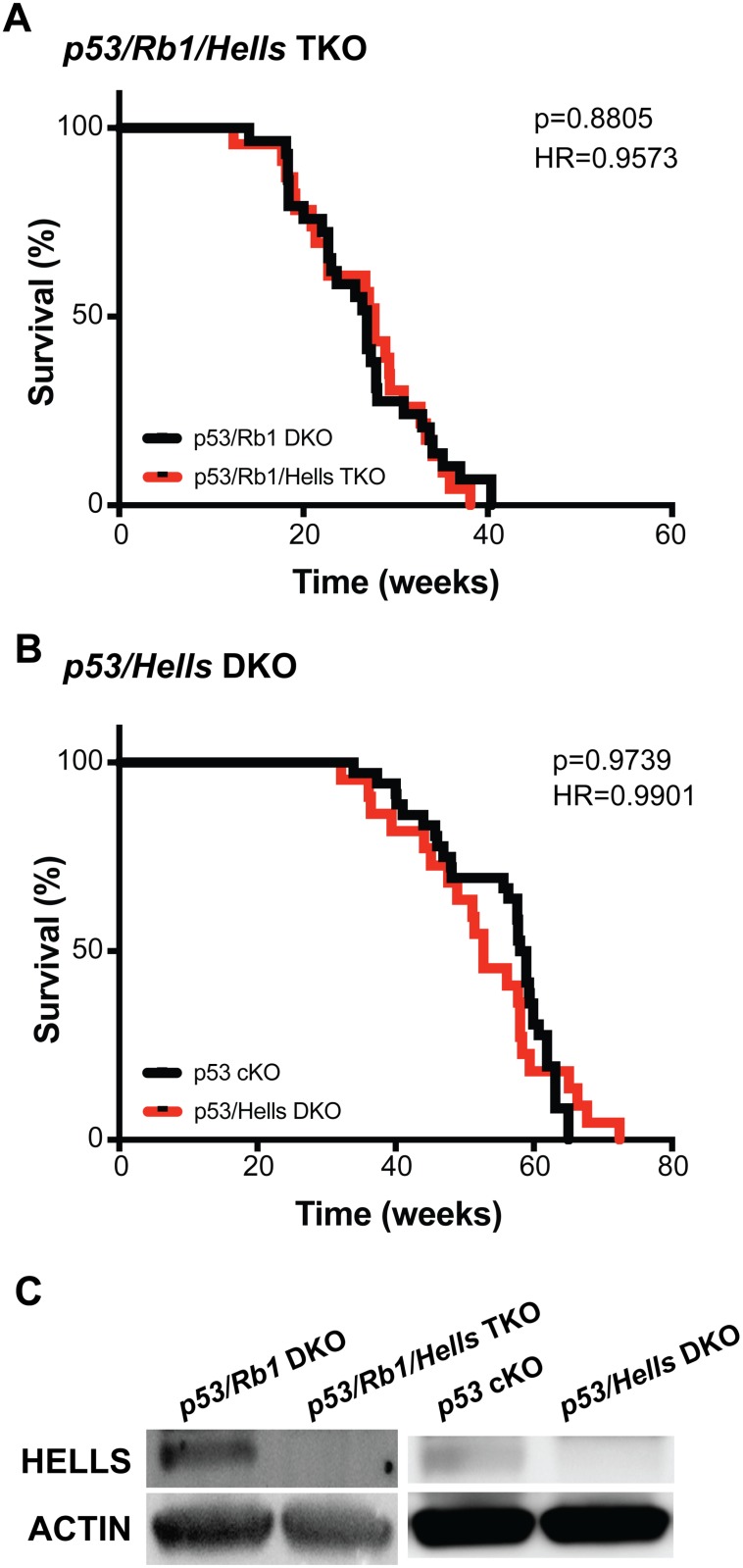
HELLS knockdown does not affect in osteosarcoma cell proliferation and migration (**A**) Western blot detection of HELLS protein level in osteosarcoma cells after transduction with shRNA against HELLS (shHELLS) compared to non-silencing controls (control) show effective decrease in HELLS protein. (**B**) Representative images from colony-forming wells with U-2OS cells transduced with shHELLS or non-silencing control. (**C**) Histogram of the proportion of number of colonies formed in osteosarcoma cells transduced with shHELLS or non-silencing control. Each bar is the mean ± s.d. of triplicate experiments. (**D**–**G**) Relative number of shHELLS and control cells over time measured by CellTiter-Glo Luminescence cell viability assay. Each data point is mean ± s.d. of four experiments. (**H**) Representative image for control (non-silencing control) and shHELLS in 143B cells. (**I**) Quantification of the distance (pixels) migrated for each of the osteosarcoma cell lines. Each data point is mean ± s.d. of ten measurements in triplicate samples. ^*^p < 0.0332 by two-tailed *t* test.

Emerging reports have linked HELLS overexpression in cancers with its ability to promote proliferation [20, 25]. To study the role of HELLS overexpression in osteosarcoma, we acquired a lentivirus encoding an shRNA sequence complimentary to HELLS (shHELLS) to facilitate HELLS gene knockdown in osteosarcoma cell lines, as well as vector-only control. Western blot analysis was used to validate successful HELLS knockdown in each osteosarcoma cell line (Figure 4A). We performed colony-forming assay on transduced osteosarcoma cell lines to assess their ability to give rise to colonies at a single-cell level. HELLS knockdown in osteosarcoma cell lines results in modest reduction (31.6–40.5%) in the ability to form colonies when compared to controls, but reached statistical significance in 143B and U-2 OS cell lines (Figure 4B–4C). Further evaluation of cellular proliferation and survival upon HELLS knockdown using CellTiter-Glo cell viability assay revealed a significant increase in cell survival and proliferation in 143B cells, but no significant change in the relative number of cells in SJSA-1, SaOS-2 and U-2 OS cells (Figure 4D–4G). This confirms the modest decreases or absence of changes observed in the colony-forming assay.

HELLS expression is positively correlated with metastatic potential, shown by increased migratory capacity of cells [27, 34]. Using scratch-wound healing assay, we assessed changes in migration potential in HELLS knockdown and control osteosarcoma cell lines. We detected no significant changes in the ability to heal the wounds within 8 h in any of the four cell lines analyzed (Figure 4H–4I).

### HELLS is dispensable for tumor initiation and progression in mouse OS

**Figure 5:**
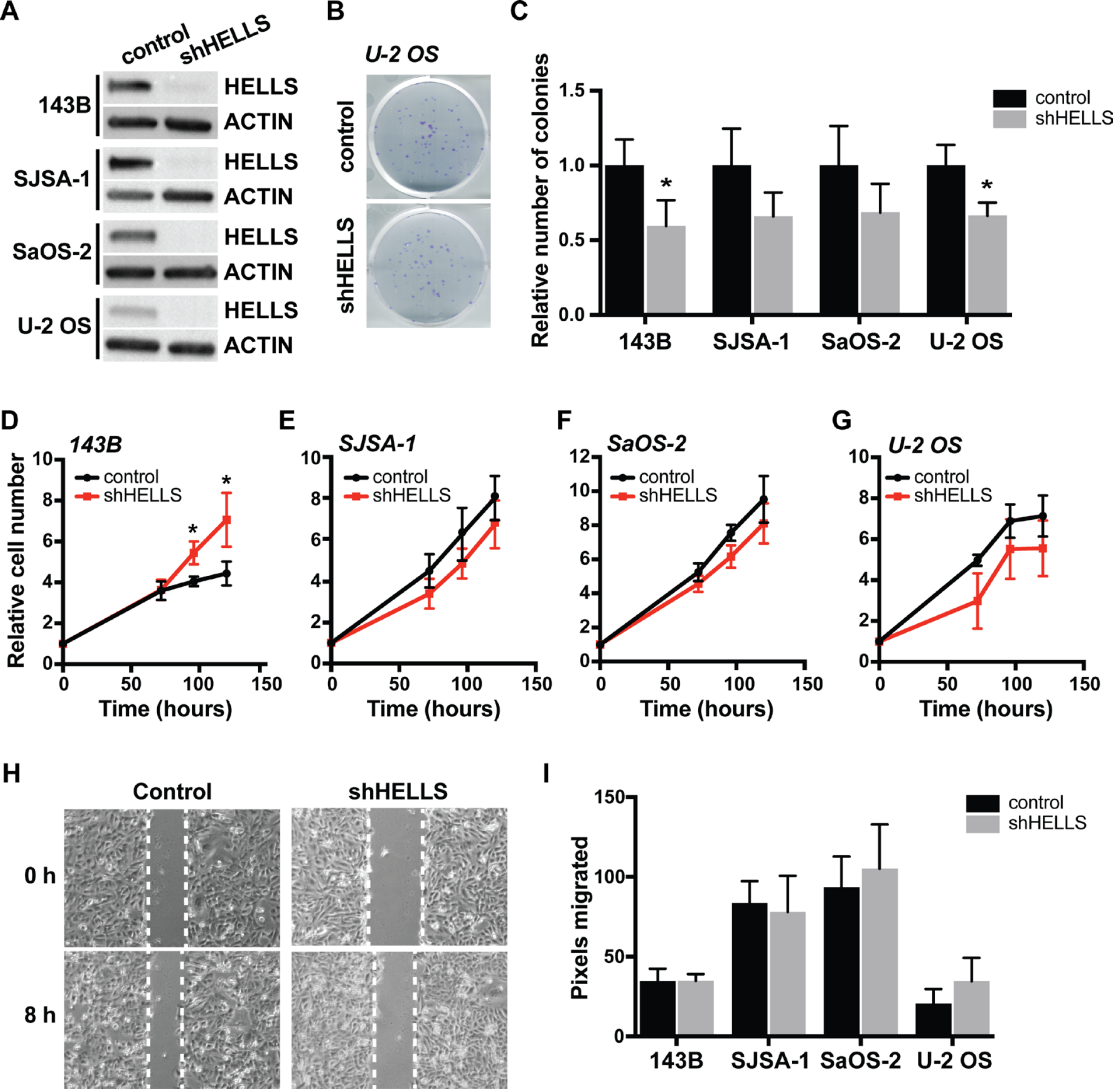
*Hells* has no effect on osteosarcoma tumorigenesis (**A**–**B**) Kaplan–Meier curves showing the survival of osteosarcoma-prone mouse models. Loss of *Hells* in osteosarcoma-prone mice show no changes in overall survival in (A) Tp53/Rb1/Hells TKO: *Osx-cre*; *Tp53^lox/lox^*; *Rb1^lox/lox^*; *Hells ^lox/lox^* (red; *n* = 23) compared to p53/Rb1 DKO: *Osx-cre*; *Tp53^lox/lox^*; *Rb1^lox/lox^* (black; *n* = 24) nor (B) p53/Hells DKO: *Osx-cre*; *p53^lox/lox^*; *Hells ^lox/lox^* (red; *n* = 22) compared to p53 cKO: Osx-cre; *p53^lox/lox^* (black; *n* = 35). Mantel-Cox test and Mantel-Haenszel hazard ratio (HR) were used for curve comparisons. (**C**) Western blot detection of HELLS confirms loss of HELLS protein in *p53/Rb1/Hells* TKO mice and *p53/Hells* DKO mice and HELLS expression in *p53/Rb1 DKO* and *p53* cKO osteosarcoma tumors.

In order to study the role of HELLS in osteosarcomagenesis, we generated genetically engineered mice to facilitate preosteoblast-specific knockout of *Hells* in two distinct genetic engineered mouse models of osteosarcoma: *p53* cKO and *p53/Rb1* DKO [28]. The resulting *p53*/*Hells* DKO (*Osx-cre p53*^lox/lox^; *Hells*^lox/lox^) and *p53*/*Rb1*/*Hells* triple knockout (TKO; *Osx-cre p53*^lox/lox^; *Rb1*^lox/lox^; *Hells*^lox/lox^) were compared to their corresponding littermate controls (*p53* cKO and *p53/Rb1* DKO, respectively) for the assessment of the role of HELLS in tumor initiation and promotion during osteosarcoma development. All genotypes lead to the development of osteosarcoma in mice with 100% penetrance. As seen in Figure 5, no differences were detected between the mice bearing *Hells* conditional knockout alleles compared to their littermate controls. The median survival in *p53/Rb1/Hells* TKO mice was 27.7 weeks (*n* = 23) compared to 26.9 weeks (*n* = 29; *p* = 0.8805) in *p53/Rb1* DKO mice (Figure 5A). The median survival in *p53/Hells* DKO mice was 52.7 weeks (*n* = 22), compared to 58.45 weeks in *p53* cKO (*n* = 35; *p* = 0.9739) (Figure 5B). Intriguingly, analysis of gender differences between the *Hells*-null mice indicated a significantly lower survival in *p53/Hells* DKO females compared to males (*p* = 0.0057; Supplementary Figure 3). Despite this difference, both males and females show no significant difference when compared to *p53* cKO mice (*p* = 0.2076 and *p* = 0.0851, respectively). Western blot analysis of *Hells* wild-type (*p53/Rb1* DKO and *p53* cKO) and *Hells*-null (*p53/Rb1/Hells* TKO and *p53/*Hells DKO) tumors confirmed that HELLS was efficiently knocked-out in these osteosarcomas (Figure 5C).

## DISCUSSION

Osteosarcoma is the most common primary cancer of bone and typically occurs in children and young adults. As a highly metastatic cancer, 15–20% of osteosarcoma patients are diagnosed after the cancer has already metastasized (typically to the lungs), which translates to 5-year survival rates of <40% [35]. Thus, there is a pressing clinical need to determine the factors responsible for metastasis in osteosarcoma to facilitate development of new therapeutic strategies. Loss of function mutations at the *RB1* gene are associated with increased mortality, metastasis and poor therapeutic outcome in osteosarcoma [4–8]. However, the mechanism(s) through which *RB1* loss leads to poor prognosis remain to be elucidated. In this study, we demonstrate that poor clinical outcome associated with loss of *RB1* is mediated, at least in part, by de-repression of aE2F family members. We also examined the role of HELLS, a chromatin remodeling protein transcriptionally regulated by E2F1 that promotes tumorigenesis in several cancers [20, 24, 27]. Here, we show that HELLS is overexpressed in osteosarcoma; however, our results indicate that while HELLS is a good biomarker for RB pathway deregulation, HELLS does not contribute to osteosarcoma tumorigenesis.

### Activator E2Fs roles in osteosarcoma

Preosteoblast-specific loss of *p53* is sufficient in driving osteosarcoma tumorigenesis in mouse [28]. Disease presentation in this model is potentiated by the additional loss of *Rb1*, which we were able to recapitulate. Here, we tried to elucidate the mechanism through which RB inactivation contributes to poor clinical outcome in osteosarcoma. We found that loss of either *E2f1* or *E2f3* significantly increased the median survival age of *p53/Rb1* DKO mice; whereas loss of *E2f5* did not improve or worsen the outcome. These results are consistent with other studies reporting that loss of transcriptional control by aE2Fs, particularly E2F1 and E2F3, is responsible for tumorigenesis following *Rb1* inactivation [20, 29, 36–38]. However, in osteosarcoma, loss of neither aE2F was sufficient to completely reverse the potentiation of the disease caused by *Rb1* loss. This could be due, at least in part, to compensation by other E2F family members (discussed in the next section). The observation that inactivation of either E2F1 or E2F3 could significantly delay the onset of tumors and increase overall survival of osteosarcoma-bearing animals suggests that the increase malignancy upon *Rb1* loss might be reversible, and that direct downstream targets of aE2Fs are valuable potential therapeutic targets.

### HELLS is transcriptionally regulated by the RB-E2F pathway

Others and we have identified HELLS as a transcriptional downstream target gene of aE2Fs that is overexpressed in cancer and contributes to tumor progression [20, 24, 27]. To determine if *HELLS* is overexpressed in osteosarcoma, we blasted The Cancer Genome Atlas (TCGA) data set and found that *HELLS* is upregulated in sarcoma tumor samples compared to normal controls. We confirmed *HELLS* gene upregulation in the human osteosarcoma cell lines 143B, SJSA-1, and SaOS-2, as well as in four of the five independent patient-derived orthotopic xenografts used in this study. Despite the lack of mRNA upregulation in some cell lines and tumors, HELLS protein overexpression was observed in all the human osteosarcoma cell lines and xenografts analyzed in this study. The discordance between mRNA and protein levels in some of the samples suggests that while HELLS protein levels are predominantly transcriptionally regulated there are also translational and post-transcriptionally mechanisms of protein expression regulation. Further, the HELLS protein overexpression observed in human osteosarcoma is recapitulated in the *p53/Rb1* DKO osteosarcoma mouse model, strengthening its validity as a study model for this disease.

Evidence of direct regulation of HELLS by the RB-E2F signaling pathway was limited to a single *in vitro* study in gliomas [24]. In this study, we confirmed that the RB-E2F signaling pathway directly regulates transcriptional activation of HELLS in the osteogenic lineage. ChIP analysis revealed enrichment of E2F1 on the promoter region of *HELLS* where putative E2F1 binding motifs reside. Interestingly, significant reduction of HELLS protein level was only achieved through combined knockdown of E2F1 and E2F3, and not by E2F1 or E2F3 knockdown alone. This suggests that E2F1 and E2F3 compensate for the loss of each other to regulate *HELLS* expression. This observation could begin to explain why losses of neither E2f1 (*p53/Rb1/E2f1* TKO) nor E2F3 (*p53/Rb1/E3f3 TKO*) were sufficient to reverse the increased malignancy observed upon *Rb1* loss (*p53/Rb1* DKO) in osteosarcoma but rather provided only partial improvements. The observation that rescue of altered expression of RB-E2F transcriptional gene targets is orchestrated by more than one E2F family member is an important consideration for therapeutic approaches being developed that aim to inhibit E2Fs directly.

RB-E2F signaling pathway is often inactivated in cancer due to various reasons including *RB1* loss, RB hyperphosphorylation through increased activity of CDK4/CDK6, or E2F hyperphosphorylation, all of which prevent repression of E2Fs from binding to downstream targets. Therefore, it is not surprising that HELLS upregulation and overexpression in osteosarcoma is not restricted to *RB1*-null cases, but rather a generalized event observed across samples that bear RB-E2F pathway inactivation. Of the osteosarcoma cell lines examined in this study, SaOS-2 harbors a deletion in the *RB1* gene that leads to a non-functional truncated protein [39], SJSA-1 has *CDK4* amplification [32], and U-2 OS is *p16* null [40]. Here, we detected upregulation of CDK4 and/or CDK6 mRNA levels in all osteosarcomas, which suggests a predominant mechanism of RB-E2F pathway inactivation through RB hyperphosphorylation in this malignancy. This was further confirmed by our observation of HELLS down-regulation upon treatment with CDK4/6 inhibitor, palbociclib.

### HELLS overexpression in osteosarcoma

The expression of HELLS has been positively associated with proliferation in both normal and malignant cells [20, 21, 27, 41]. Multiple studies have shown that HELLS knockdown *in vitro* results in decreased proliferation of various cancer cell types including retinoblastoma [20], gliomas [24], nasopharyngeal [27] and breast cancer [34] cells, and that engraftment of cells carrying ectopically expressed HELLS gives rise to heavier tumor burden. In some cases, HELLS expression is positively correlated with metastatic potential, shown by increased migratory capacity of cells and promotion of epithelial to mesenchymal transition [27, 34]. In this study, we demonstrated that HELLS knockdown in osteosarcoma cell lines modestly decreased colonizing capacity and had no effect on proliferation, except in 143B cells where we found increased proliferative capacity, opposite of expected. Further, we found no effect of HELLS on migratory potential in osteosarcoma.

Beyond *in vitro* loss-of-function studies in human osteosarcoma models, we generated novel genetic engineered mouse models to study the role of *Hells* in osteosarcoma tumor formation. We observed that *p53/Hells* DKO and *p53/Rb1/Hells* TKO mice showed no improvement in tumor incidence or overall survival compared to *p53* cKO and *p53/Rb1* DKO controls, respectively. This result suggests that HELLS is not a critical driver of *Rb1*-mediated malignancy in murine osteosarcoma tumors.

Taken together, our studies and others indicate that HELLS may be a reliable biomarker for RB-E2F pathway inactivation. However, HELLS upregulation may not always be synonymous to a critical role in tumorigenesis or tumor progression, or a warranted target for therapeutics in all malignancies where HELLS overexpression is observed.

## MATERIALS AND METHODS

### Xenografts, mouse models of osteosarcoma and cell lines

The five orthotopic xenografts used in this study: SJOS001105 (PDX1), SJOS001112 (PDX2), SJOS001107 (PDX3), SJSO010930 (PDX4), and SJOS001121 (PDX5) were obtained from the Childhood Solid Tumor Network [42]. Athymic nude (NU/J) mice were obtained from The Jackson Laboratories.

The *p53^lox/lox^* and *Rb1^lox/lox^* conditional-knockout mice were obtained from the Mouse Models of Human Cancer Consortium at the National Cancer Institute; *E2f1*-knockout and *E2f3^lox/lox^* conditional knockout mice were obtained from Dr. Gustavo Leone (The Ohio State University); *E2f5^lox/lox^* mice were obtained from Dr. Joseph Nevins (Duke University); the *Osx-cre* mice were obtained from The Jackson Laboratory. The *Hells*-conditional knockout mouse was generated from mice obtained from the European Mouse Mutant Archive, backcrossed to Flp mice for removal of the neo-cassette (tm1c conversion), and then backcrossed to C57BL/6N mice for Flp removal. Mice were monitored weekly for signs of osteosarcoma. Moribund status was defined as the point when tumors had reached 10% body weight or induced paralysis in the mouse. The University of California Irvine Institutional Animal Care and Use Committee approved all animal procedures. Survival curves were generated using GraphPad Prism. Mantel-Cox test was used for statistical analyzes of the curves.

Osteosarcoma cell lines 143B, SJSA-1, SaOS-2 and U-2 OS, as well as MSCs and HEK293T cells were acquired from the American Type Culture Collection (ATCC). Cells were cultured in a humidified atmosphere at 37° C and 5% CO_2_. 143B were cultured in MEM (Gibco), with 10% BCS, penicillin/streptomycin, and 0.015 mg/ml BrdU. SJSA-1 were cultured in RPMI (Gibco), with 10% BCS and penicillin/streptomycin. SaOS-2 were cultured in McCoy's 5A (Gibco), with 15% BCS and penicillin/streptomycin. U-2 OS were cultured in McCoy's 5A (Gibco), with 10% BCS and penicillin/streptomycin. MSCs were cultured in Alpha-MEM (Gibco), with 10% FBS and penicillin/streptomycin. HEK293T cells were culture in high-glucose DMEM (Gibco), with 10% BCS, penicillin/streptomycin and sodium pyruvate. Cells were passaged every 3 to 4 days or when they reached 70–80% confluency. At the time of passage, cells were split to 20% confluence.

### Real-time RT-PCR

Total RNA was isolated from cells or tumor tissues by homogenizing samples with TRIzol Reagent. RNA was isolated through chloroform extraction. 1 μg of total RNA was used for cDNA synthesis with the SuperScript™III First-strand synthesis system (Invitrogen) according to manufacturer's protocol at a reaction volume of 20 μl. Quantitative PCR amplification was performed using 1 μl of reverse-transcribed product in Power SYBR Green PCR Master Mix (4367659, Life Technologies). Primers were designed using IDT Real-Time PCR tool (Integrated DNA Technologies). Reaction was carried out using 7500 Real-Time PCR system (Applied Biosciences). Data were normalized to those obtained with endogenous control 18S and GAPDH mRNA, and analyzed using ΔΔ*C*_t_ method. Primer sequence for PCR amplification are as follows: HELLS (Forward 5′-ACAGGCTGATGTGTACTTAACC-3′; Reverse 5′- TCCCCATGAAAAGCCTACTTC-3′), CDK4 (Forward 5′-ACACTGAGAGCGCAATCTTTG-3′; Reverse 5′-GAG AAATGGGAAGGAGAAGGAG-3′), CDK6 (Forward 5′-AAAGTGTTCCCTGCTACCATC-3′; Reverse 5′-CAG CATCAGGAACCATCTCTAG-3′), GAPDH (Forward: 5′-AGCAAGAGCACAAGAGGAAG-3′; Reverse: 5′-TCT ACATGGCAACTGTGAGG-3′), 18S (Forward 5′-GTA ACCCGTTGAACCCCATT-3′; Reverse 5′-CCATCCAA TCGGTAGTAGCG-3′).

### Western blotting

Cell pellets or tumor tissues were homogenized by pipetting or pellet pestle in RIPA buffer (50 mM Tris-HCl, pH = 8, 150 mM NaCl, 1% NP-40, 0.5% Sodium deoxycholate, 0.1% SDS, 1 mM EDTA) supplemented with protease inhibitor (cOmplete™, Mini, EDTA-free Protease Inhibitor Cocktail, Roche). Samples were allowed to lyse for 30 min on ice before centrifugation at 14000 RPM at 4° C for 30 min. Protein concentration was measured using BCA protein assay (Pierce™ BCA Protein Assay Kit). 40 μg of total protein were resolved in 4–15% SDS-PAGE gel (Mini-PROTEAN TGX Gels (4–15%), Bio-rad), and transferred onto PVDF membrane (Immobilon-P Membrane, PVDF, EMD Millipore) using semi-dry transfer apparatus (Bio-rad). Ponceau S stain was used to validate successful transfer. Non-specific binding was prevented by incubating the membrane in 3% non-fat dry milk in TBS-0.25% Tween (TBS-T) for 1 hour at room temperature, shaking. Primary antibodies were diluted in 0.5% non-fat dry milk in TBS-T as follows: 1:1000 anti-HELLS (sc-28202, Santa Cruz Biotechnology), anti-actin (A1978, Sigma), anti-E2F1 (3742S, Cell Signaling), anti-E2F2 (sc-9967, Santa Cruz Biotechnology), anti-E2F3 (MA5-11319, Nalgene Nunc), 1:1000 anti-Rb (9313T, Cell Signaling). Membranes were incubated in primary antibody overnight, shaking, at 4° C. Membranes were then rinsed 3 times with TBS-T on shaker and incubated with secondary antibody for 40min at room temperature, shaking. Secondary antibodies were diluted in 0.5% non-fat dry milk in TBS-T as follows: 1:1000 peroxidase labeled anti-mouse IgG (PI-2000, Vector Laboratories), 1:1000 peroxidase labeled anti-rabbit IgG (PI-1000, Vector Laboratories). Membranes were again rinsed 3 times with TBS-T on shaker. Chemiluminescence was detected using SuperSignal™ West Pico Chemiluminescent Substrate (34077, Thermo Scientific). Relative band intensity was analyzed using ImageJ software.

### Chromatin immunoprecipitation (ChIP assay)

ChIP assays were essentially performed as previously described [20, 43]. ChIP DNA was analyzed by qPCR with SYBR Green (Bio-Rad) in ABI-7500 (Applied Biosystems) using the following primers: Forward: 5′-CCTGAGAGAGGTCCAGGTAAA-3′; Reverse 5′-CTGTCATCTCGCGATACCTAAC-3′. The antibodies used were anti-E2F1 (3742; Cell Signaling) and rabbit IgG (sc-2027, Santa Cruz Biotechnologies).

### Palbociclib treatment

Osteosarcoma cells were seeded at a density of 500000 cells/well of 6-well plate and allowed to adhere overnight. Two concentrations of Palbociclib, 0.5 μM and 1 μM, were used to treat cells for 24 h prior to collection. DMSO was used as drug vehicle control.

### Lentivirus production and transduction

Lentiviral particles were produced by co-transfecting the envelope plasmid pCMV-VSV-G (Addgene), packaging plasmid pCMV-dR8.2 dvpr (Addgene), and GIPZ shRNA vectors (GE Dharmacon): E2F1 shRNA (V3LHS_393591), E2F2 shRNA (V3LHS_324068), E2F3 shRNA (V3LHS_325936), HELLS shRNA (V2LHS_155497), or GIPZ lentiviral empty vector shRNA control into HEK293T cells using calcium phosphate transfection method. Supernatants containing lentiviral particles were harvested at 24 and 48 hours post-transfection. Cell debris were cleared by centrifugation at 1600 × g for 10 min at 4° C. Supernatants were then filtered through 0.45μm PES filter (25–223, Genesee Scientific), and concentrated by ultracentrifugation at 23000 RPM for 2 hours at 4° C. Lentiviral particles were resuspended in ice-cold PBS and stored at −80° C. Transduction of target cells were achieved by exposing cells to viral particles in serum-free condition for 6 hours. Puromycin selection was carried out at a concentration of 2 μg/ml.

### Cell viability assay

All cells were seeded into 96-well black assay plates (Costar) at a density of 3,000 cells per well. Cell viability was determined at 24, 72, and 96 hours after seeding using CellTiter-Glo (Promega) according to manufacturer's instructions. Luminescence readings were normalized to the 24 h post-seeding reading. Survival curves were generated using GraphPad Prism.

### Colony formation assay

Lentiviral transduced cells were seeded at a low-density (20 cells/cm^2^) as single cells onto 6-well cell culture plates in the appropriate cell culture medium. Fresh medium was supplemented every 4 days. Cells were incubated at 37° C with 5% CO_2_ until cells in control dishes have formed sufficiently large colonies (~50 cells). Cells were washed once in PBS before fixing and staining in a mixture consisting of 6% glutaraldehyde and 0.5% crystal violet for 30 min. Stains were washed out with tap water and the plates left to dry at room temperature. Colonies were counted from triplicate experiments.

### Scratch-wound healing assay

Scratch-wound assay was performed as previously described [44]. Cells (1 × 10^6^/well) were plated on 6-well plates. The day after, once cells were grown to a confluence of about 80%, the monolayer was scratched using a 10 μl sterile pipette tip. Images were captured immediately and 8 h after the wound. The exact location of the image was marked to identify the same gap. The distances between the boundaries of the wound at 0 and 8 h at 10 different locations were measured in pixels using Zen software (Zeiss).

## SUPPLEMENTARY MATERIALS FIGURES AND TABLES



## References

[1] Geller DS, Gorlick R (2010). Osteosarcoma: a review of diagnosis, management, and treatment strategies. Clin Adv Hematol Oncol.

[2] Martin JW, Squire JA, Zielenska M (2012). The genetics of osteosarcoma. Sarcoma.

[3] Chen X, Bahrami A, Pappo A, Easton J, Dalton J, Hedlund E, Ellison D, Shurtleff S, Wu G, Wei L, Parker M, Rusch M, Nagahawatte P, St. Jude Children’s Research Hospital–Washington University Pediatric Cancer Genome Project (2014). Cell Rep.

[4] Feugeas O, Guriec N, Babin-Boilletot A, Marcellin L, Simon P, Babin S, Thyss A, Hofman P, Terrier P, Kalifa C, Brunat-Mentigny M, Patricot LM, Oberling F (1996). Loss of heterozygosity of the RB gene is a poor prognostic factor in patients with osteosarcoma. J Clin Oncol.

[5] Benassi MS, Molendini L, Gamberi G, Sollazzo MR, Ragazzini P, Merli M, Magagnoli G, Sangiorgi L, Bacchini P, Bertoni F, Picci P (1997). Altered G1 phase regulation in osteosarcoma. Int J Cancer.

[6] Goto A, Kanda H, Ishikawa Y, Matsumoto S, Kawaguchi N, Machinami R, Kato Y, Kitagawa T (1998). Association of loss of heterozygosity at the p53 locus with chemoresistance in osteosarcomas. Jpn J Cancer Res.

[7] Wadayama B, Toguchida J, Shimizu T, Ishizaki K, Sasaki MS, Kotoura Y, Yamamuro T (1994). Mutation spectrum of the retinoblastoma gene in osteosarcomas. Cancer Res.

[8] Ren W, Gu G (2017). Prognostic implications of RB1 tumour suppressor gene alterations in the clinical outcome of human osteosarcoma: a meta-analysis. Eur J Cancer Care (Engl).

[9] Luetke A, Meyers PA, Lewis I, Juergens H (2014). Osteosarcoma treatment - where do we stand? A state of the art review. Cancer Treat Rev.

[10] Ottaviani G, Jaffe N (2009). The epidemiology of osteosarcoma. Cancer Treat Res.

[11] Damron TA, Ward WG, Stewart A (2007). Osteosarcoma, chondrosarcoma, and Ewing’s sarcoma: National Cancer Data Base Report. Clin Orthop Relat Res.

[12] Kaste SC, Pratt CB, Cain AM, Jones-Wallace DJ, Rao BN (1999). Metastases detected at the time of diagnosis of primary pediatric extremity osteosarcoma at diagnosis: Imaging features. Cancer.

[13] DeGregori J, Johnson DG (2006). Distinct and Overlapping Roles for E2F Family Members in Transcription, Proliferation and Apoptosis. Curr Mol Med.

[14] Harbour JW, Dean DC (2000). Chromatin remodeling and Rb activity. Curr Opin Cell Biol.

[15] Morris EJ, Dyson NJ (2001). Retinoblastoma protein partners. Adv Cancer Res.

[16] Talluri S, Dick FA (2012). Regulation of transcription and chromatin structure by pRB: here, there and everywhere. Cell Cycle.

[17] Kansara M, Thomas DM (2007). Molecular pathogenesis of osteosarcoma. DNA Cell Biol.

[18] Benassi MS, Molendini L, Gamberi G, Ragazzini P, Sollazzo MR, Merli M, Asp J, Magagnoli G, Balladelli A, Bertoni F, Picci P (1999). Alteration of pRb/p16/cdk4 regulation in human osteosarcoma. Int J Cancer.

[19] Maitra A, Roberts H, Weinberg AG, Geradts J (2001). Loss of p16(INK4a) expression correlates with decreased survival in pediatric osteosarcomas. Int J Cancer.

[20] Benavente CA, Finkelstein D, Johnson DA, Marine JC, Ashery-Padan R, Dyer MA (2014). Chromatin remodelers HELLS and UHRF1 mediate the epigenetic deregulation of genes that drive retinoblastoma tumor progression. Oncotarget.

[21] Geiman TM, Muegge K (2000). Lsh, an SNF2/helicase family member, is required for proliferation of mature T lymphocytes. Proc Natl Acad Sci U S A.

[22] Tao Y, Xi S, Shan J, Maunakea A, Che A, Briones V, Lee EY, Geiman T, Huang J, Stephens R, Leighty RM, Zhao K, Muegge K (2011). Lsh, chromatin remodeling family member, modulates genome-wide cytosine methylation patterns at nonrepeat sequences. Proc Natl Acad Sci U S A.

[23] Chen D, Maruschke M, Hakenberg O, Zimmermann W, Stief CG, Buchner A (2017). TOP2A, HELLS, ATAD2, and TET3 Are Novel Prognostic Markers in Renal Cell Carcinoma. Urology.

[24] Xiao D, Huang J, Pan Y, Li H, Fu C, Mao C, Cheng Y, Shi Y, Chen L, Jiang Y, Yang R, Liu Y, Zhou J (2017). Chromatin Remodeling Factor LSH is Upregulated by the LRP6-GSK3beta-E2F1 Axis Linking Reversely with Survival in Gliomas. Theranostics.

[25] von Eyss B, Maaskola J, Memczak S, Mollmann K, Schuetz A, Loddenkemper C, Tanh MD, Otto A, Muegge K, Heinemann U, Rajewsky N, Ziebold U (2012). The SNF2-like helicase HELLS mediates E2F3-dependent transcription and cellular transformation. EMBO J.

[26] Ryu B, Kim DS, Deluca AM, Alani RM (2007). Comprehensive expression profiling of tumor cell lines identifies molecular signatures of melanoma progression. PLoS One.

[27] He X, Yan B, Liu S, Jia J, Lai W, Xin X, Tang CE, Luo D, Tan T, Jiang Y, Shi Y, Liu Y, Xiao D (2016). Chromatin Remodeling Factor LSH Drives Cancer Progression by Suppressing the Activity of Fumarate Hydratase. Cancer Res.

[28] Walkley CR, Qudsi R, Sankaran VG, Perry JA, Gostissa M, Roth SI, Rodda SJ, Snay E, Dunning P, Fahey FH, Alt FW, McMahon AP, Orkin SH (2008). Conditional mouse osteosarcoma, dependent on p53 loss and potentiated by loss of Rb, mimics the human disease. Genes Dev.

[29] Sangwan M, McCurdy SR, Livne-Bar I, Ahmad M, Wrana JL, Chen D, Bremner R (2012). Established and new mouse models reveal E2f1 and Cdk2 dependency of retinoblastoma, and expose effective strategies to block tumor initiation. Oncogene.

[30] Daily K, Patel VR, Rigor P, Xie X, Baldi P (2011). MotifMap: integrative genome-wide maps of regulatory motif sites for model species. BMC Bioinformatics.

[31] Wei G, Lonardo F, Ueda T, Kim T, Huvos AG, Healey JH, Ladanyi M (1999). CDK4 gene amplification in osteosarcoma: reciprocal relationship with INK4A gene alterations and mapping of 12q13 amplicons. Int J Cancer.

[32] Al-Assar O, Rees-Unwin KS, Menasce LP, Hough RE, Goepel JR, Hammond DW, Hancock BW (2006). Transformed diffuse large B-cell lymphomas with gains of the discontinuous 12q12-14 amplicon display concurrent deregulation of CDK2, CDK4 and GADD153 genes. Br J Haematol.

[33] Fry DW, Harvey PJ, Keller PR, Elliott WL, Meade M, Trachet E, Albassam M, Zheng X, Leopold WR, Pryer NK, Toogood PL (2004). Specific inhibition of cyclin-dependent kinase 4/6 by PD 0332991 and associated antitumor activity in human tumor xenografts. Mol Cancer Ther.

[34] Tao Y, Liu S, Briones V, Geiman TM, Muegge K (2011). Treatment of breast cancer cells with DNA demethylating agents leads to a release of Pol II stalling at genes with DNA-hypermethylated regions upstream of TSS. Nucleic Acids Res.

[35] Ottaviani G, Jaffe N (2009). The epidemiology of osteosarcoma. Cancer Treat Res.

[36] Yamasaki L, Bronson R, Williams BO, Dyson NJ, Harlow E, Jacks T (1998). Loss of E2F-1 reduces tumorigenesis and extends the lifespan of Rb1(+/-)mice. Nat Genet.

[37] Ziebold U, Lee EY, Bronson RT, Lees JA (2003). E2F3 loss has opposing effects on different pRB-deficient tumors, resulting in suppression of pituitary tumors but metastasis of medullary thyroid carcinomas. Mol Cell Biol.

[38] Parisi T, Yuan TL, Faust AM, Caron AM, Bronson R, Lees JA (2007). Selective requirements for E2f3 in the development and tumorigenicity of Rb-deficient chimeric tissues. Mol Cell Biol.

[39] Shew JY, Lin BT, Chen PL, Tseng BY, Yang-Feng TL, Lee WH (1990). C-terminal truncation of the retinoblastoma gene product leads to functional inactivation. Proc Natl Acad Sci U S A.

[40] Gump J, Stokoe D, McCormick F (2003). Phosphorylation of p16INK4A correlates with Cdk4 association. J Biol Chem.

[41] Han Y, Ren J, Lee E, Xu X, Yu W, Muegge K (2017). Lsh/HELLS regulates self-renewal/proliferation of neural stem/progenitor cells. Sci Rep.

[42] Stewart E, Federico S, Karlstrom A, Shelat A, Sablauer A, Pappo A, Dyer MA (2016). The Childhood Solid Tumor Network: A new resource for the developmental biology and oncology research communities. Dev Biol.

[43] Benavente CA, McEvoy JD, Finkelstein D, Wei L, Kang G, Wang YD, Neale G, Ragsdale S, Valentine V, Bahrami A, Temirov J, Pounds S, Zhang J, Dyer MA (2013). Cross-species genomic and epigenomic landscape of retinoblastoma. Oncotarget.

[44] Zocchi L, Wu SC, Wu J, Hayama KL, Benavente CA (2018). The cyclin-dependent kinase inhibitor flavopiridol (alvocidib) inhibits metastasis of human osteosarcoma cells. Oncotarget.

