# Supplementary material for: Chromatin remodeling protein HELLS is upregulated by inactivation of the RB-E2F pathway and is nonessential for osteosarcoma tumorigenesis
Source: Oncotarget. 2018 Aug 24;9(66):32580–92. doi: 10.18632/oncotarget.25953 (PMC6135688; doi:10.18632/oncotarget.25953)
Supplement: Supplementary file 1 [file oncotarget-09-32580-s001.pdf]

Chromatin remodeling protein HELLS is upregulated by inactivation of the RB-E2F pathway and is nonessential for osteosarcoma tumorigenesis

SUPPLEMENTARY MATERIALS

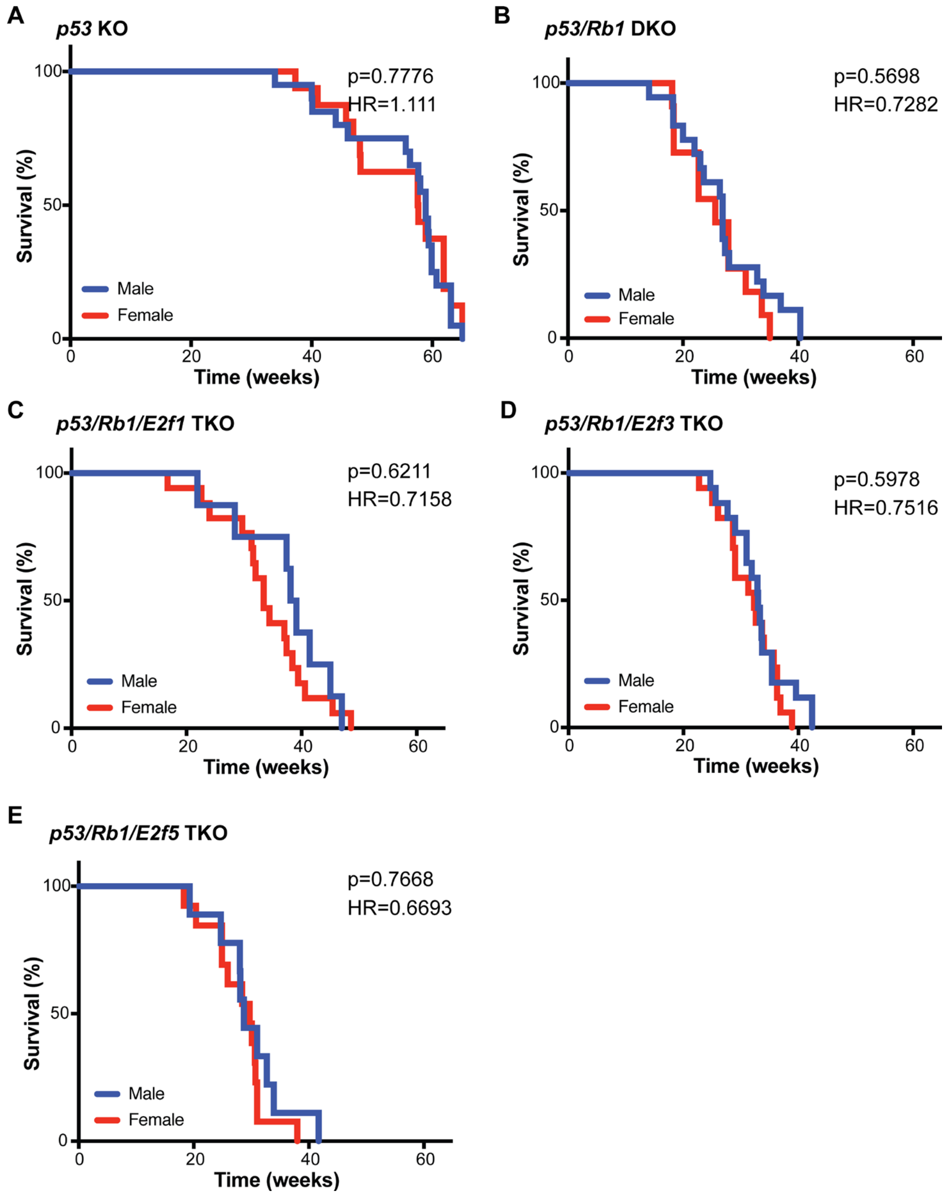

Supplementary Figure 1: Osteosarcoma mouse models display no gender variation. Kaplan–Meier curves showing the survival of osteosarcoma-prone mouse models divided by gender. (**A**) p53 cKO: *Osx-cre*; $p53^{lox/lox}$ (male: *n* = 19; female: *n* = 16). (**B**) p53/Rb1 DKO: *Osx-cre*; $Tp53^{lox/lox}$; $Rb1^{lox/lox}$ (male: *n* = 18; female: *n* = 11). (**C**) *p53/Rb1/E2f1* TKO: *Osx-cre*; $p53^{lox/lox}$; $Rb1^{lox/lox}$; $E2f1^{-/-}$ (male: *n* = 8; female: *n* = 17). (**D**) *p53/Rb1/E2f3* TKO: *Osx-cre*; $p53^{lox/lox}$;$Rb1^{lox/lox}$;$E2f3^{lox/lox}$ (male: *n* = 17; female: *n* = 17). (**E**) *p53/Rb1/E2f5* TKO: *Osx-cre*; $p53^{lox/lox}$;$Rb1^{lox/lox}$;$E2f5^{lox/lox}$ (male: *n* = 9; female: *n* = 13). Mantel-Cox test and Mantel-Haenszel hazard ratio (HR) were used for curve comparisons.

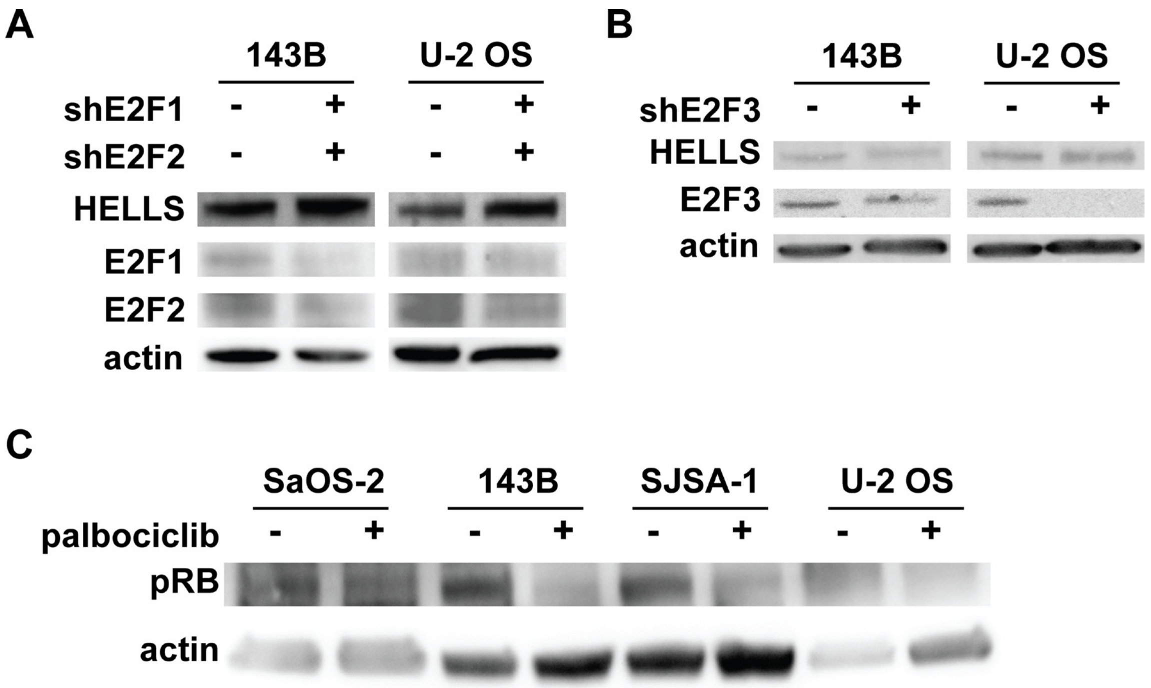

Supplementary Figure 2: E2F2 or E2F3 knockdown does not affect HELLS protein levels and palbociclib decreases phosphorylated RB. Western blot detection of (**A**) E2F1, E2F2 and HELLS protein level in osteosarcoma cells transduced with lentivirus containing shRNA against E2F1 (shE2F1) and E2F2 (shE2F2) show no significant change in HELLS expression; (**B**) E2F3 and HELLS protein level in osteosarcoma cells transduced with lentivirus containing shRNA against E2F3 (shE2F3) show no significant change in HELLS expression, and (**C**) phosphorylated RB (pRB) in osteosarcoma cell lines after Palbociclib-treated for 24 h show decreased phosphor-RB, Actin used as loading control.

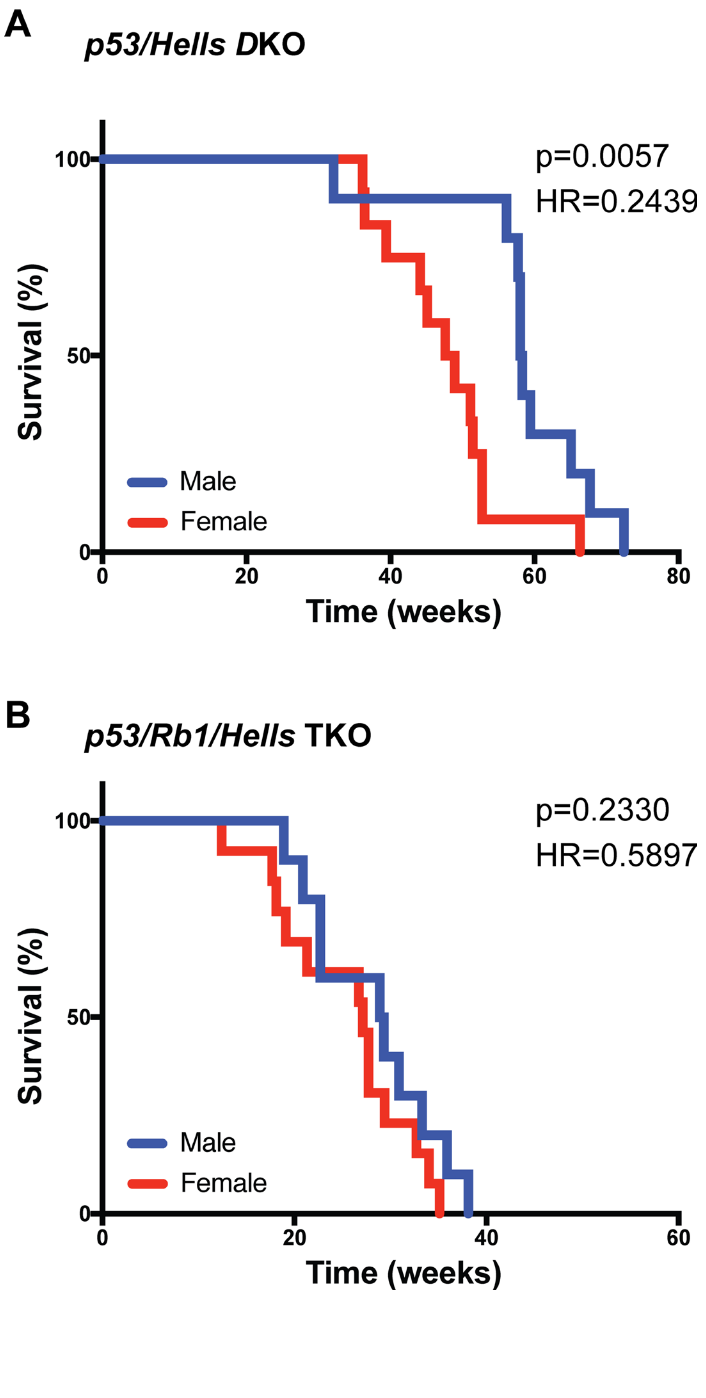

Supplementary Figure 3: Males present a better prognosis in p53/Hells DKO mice. Kaplan–Meier curves showing the survival of *Hells*-null osteosarcoma mouse models divided by gender. (**A**) p53/Hells DKO: *Osx-cre*; *Tp53*$^{lox/lox}$; *Hells*$^{lox/lox}$ (male: n = 10; female: n = 13). (**B**) Tp53/Rb1/Hells TKO: *Osx-cre*; *Tp53*$^{lox/lox}$; *Rb1*$^{lox/lox}$; *Hells* $^{lox/lox}$ (male: n = 10; female: n = 12). Mantel-Cox test and Mantel-Haenszel hazard ratio (HR) were used for curve comparisons.
